# Oral erythema multiforme after Pfizer-BioNTech COVID-19 vaccination: a report of four cases

**DOI:** 10.1186/s12903-022-02124-2

**Published:** 2022-03-24

**Authors:** Massimo Petruzzi, Sara Galleggiante, Sabrina Messina, Fedora della Vella

**Affiliations:** 1 Interdisciplinary Department of Medicine, University of Bari “Aldo Moro”, Bari, Italy; 2 Department of Restorative, Preventive and Pediatric Dentistry, University of Bern, Bern, Switzerland

**Keywords:** Covid-19 vaccine, BioNTech/Pfizer, Erythema multiforme, Adverse reaction, Case report

## Abstract

**Background:**

The 2019 Coronavirus disease (Covid-19) has affected thousands of people worldwide. To date, vaccines appear to be the only method to prevent and reduce mortality. Four vaccinations have been outwardly approved by European Medicine Agency (EMA) in Europe: BNT162b2 (Comirnaty-BioNTech/Pfizer), mRNA-1273 (Spikevax-Moderna), ChAdOx1 (VaxzevriaAstrazeneca), and Ad26.COV2-S (Janssen-Johnson&Johnson). After vaccination, local and systemic adverse effects can occur. Cutaneous reactions like urticaria, local injection site pain, morbilliform rash have been documented after vaccination.

**Cases presentation:**

We report four cases of oral erythema multiforme flare arising after BNT162b2 vaccination administration. All the patients denied previous erythema-like and herpetic manifestations history. Two of the reported cases (number 1 and 2) presented with both oral and cutaneous lesions, while cases 3 and 4 showed only oral manifestations. Three of the cases presented the erythema after the first vaccination dosage administration, only one case reported lesions after the second vaccination dosage administration. All the cases were treated with prednisone via oral administration and topical 0.05% clobetasol ointment.

**Conclusions:**

The present reports represent some of the few cases of erythema multiforme occurring as a side effect of the BNT162b2 COVID-19 vaccination. The causal role of the vaccine for the erythema multiforme has not been proven yet; nevertheless, it is not uncommon for medications to trigger this disease. The vaccine could surface a silent herpes virus infection, which would induce the erythema multiforme instead.

## Background

The 2019 coronavirus disease (Covid-19) has affected thousands of people worldwide. Eighty percent of them were hospitalized and 7.4% died from complications of infection [1].

At first, it was considered only a respiratory disease, afterward, it was found that Covid-19 can cause a large range of complications and systemic involvement (thromboembolic, gastrointestinal, cardiovascular, immune, renal, and neurological) that can lead to multiple organ dysfunction [1].

Nevertheless, oral signs like dry mouth, dysgeusia, candidiasis, ulcerations, herpetic lesions, muscle pain and swelling are reported in several cases of Covid-19 [2].

To date, vaccines appear to be the only method to prevent and reduce mortality, other than lightening the burden on healthcare systems [3]. Four vaccinations have been outwardly cleared by European Medicine Agency (EMA) in Europe: BNT162b2 (Comirnaty-BioNTech/Pfizer), mRNA-1273 (Spikevax-Moderna), ChAdOx1 (Vaxzevria-AstraZeneca), and Ad26.COV2-S (Janssen-Johnson&Johnson). Others vaccines are under evaluation for approval.

After BNT162b2 administration, local and systemic adverse effects can occur, rarely they can be severe. The most common cutaneous reactions reported after vaccination are urticaria (4.8–26%), local injection site reaction/pain (24–54%), and morbilliform rash (4.1–18%) [4].

Moreover, 5 varicella-zoster virus reactivation cases after BNT162b2 vaccination were described [5]. We report the case of 4 patients developing oral mucosal flare erythema multiforme (EM) after the BNT162b2 vaccine administration.

## Cases presentation

### Case 1

A 55-year-old female presented at the Oral Medicine section of Policlinic of Bari complaining of painful lesions on lips, oral mucosa, hands, knees, and feet. A year earlier, she came to our attention due to desquamative gingivitis, which was thereafter diagnosed as Mucous Membrane Pemphigoid (MMP). After corticosteroid-based therapy, the MMP remitted for 11 months.

Twenty-four hours after receiving the first dose of vaccine administration, she did not report any symptoms, except mild pain on the injection site.

Ten days after, the patient reported the development of spread squamous crusted lesions on the labial skin and vermillion.

**Fig. 1:**
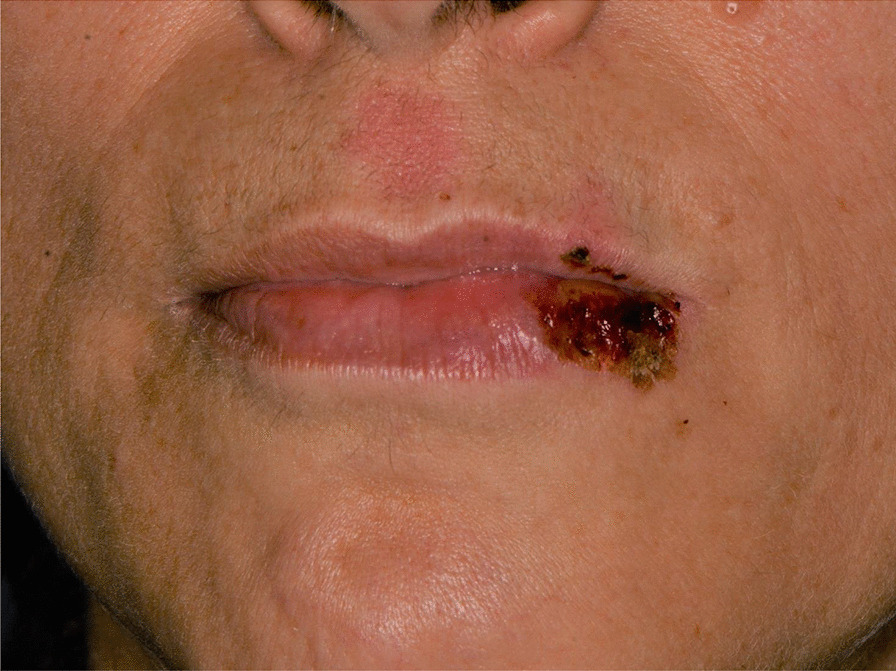
Squamous crusted lesions and erythematous areas on lips mucosa and vermilion

**Fig. 2:**
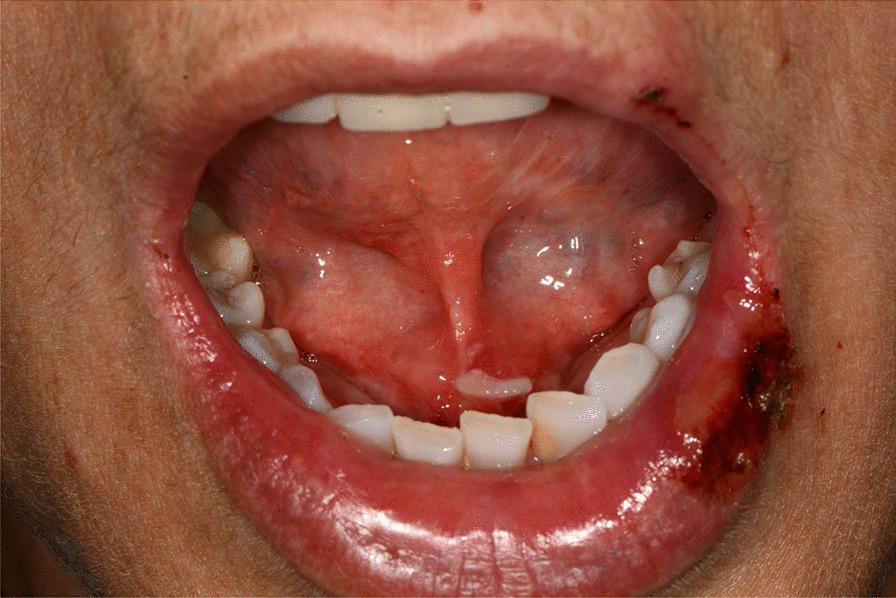
Squamous crusted lesions on lips mucosa and bullous lesions on the mouth floor mucosa

**Fig. 3:**
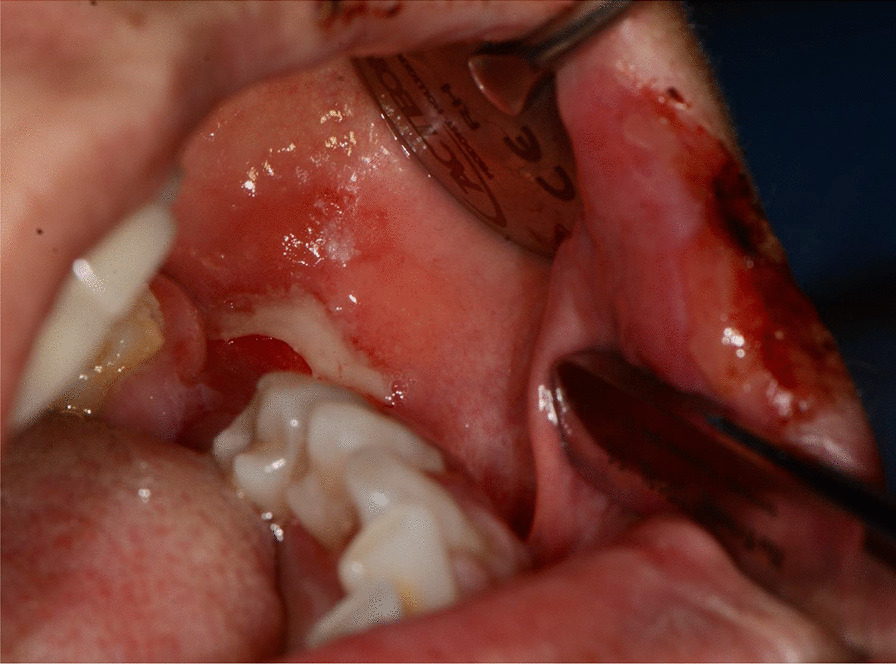
Bullous-erythematosus lesions on the left cheek mucosa

**Fig. 4:**
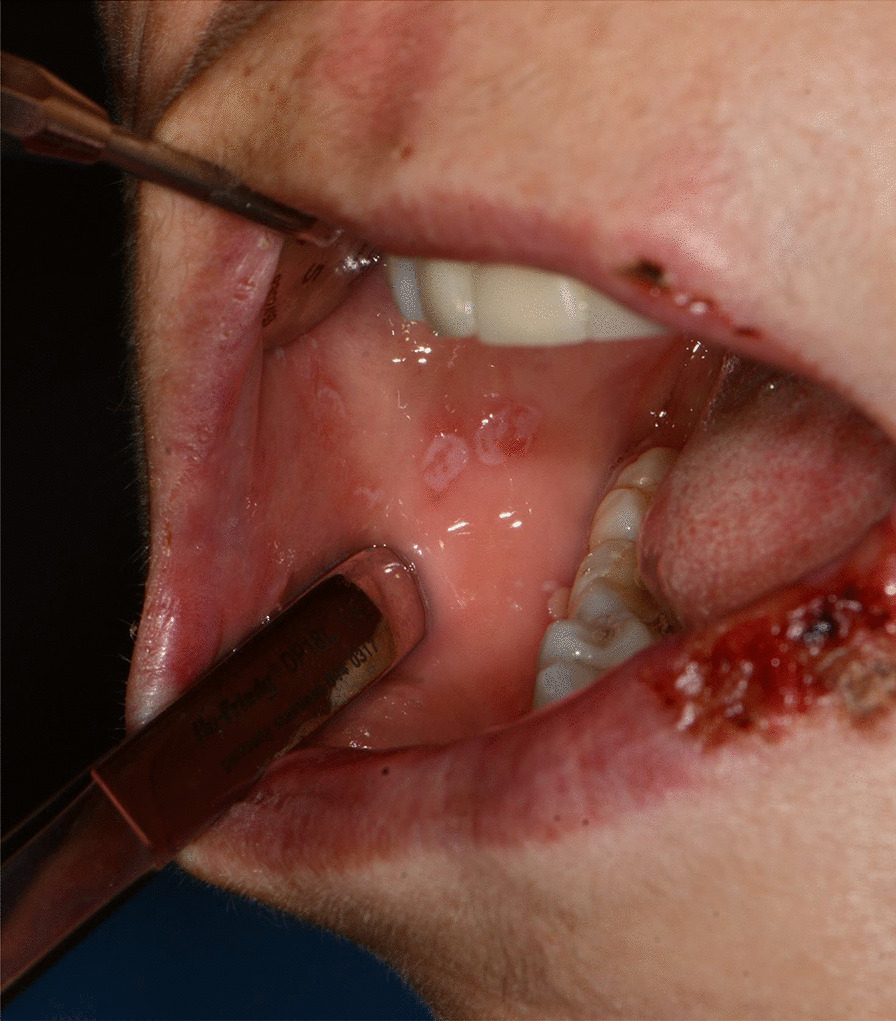
Bullous-erythematosus lesions on the right cheek mucosa

**Fig. 5:**
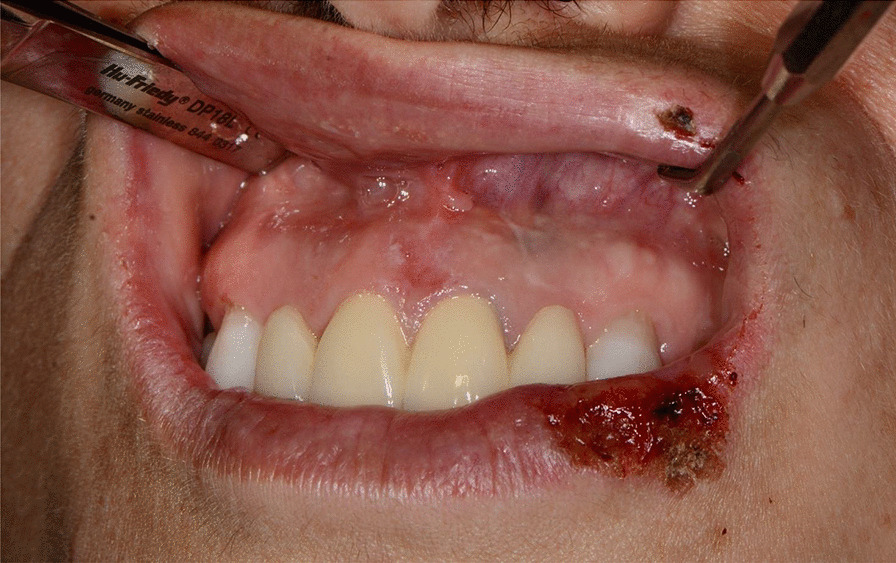
Erythematous lesions on the gingival mucosa

**Fig. 6:**
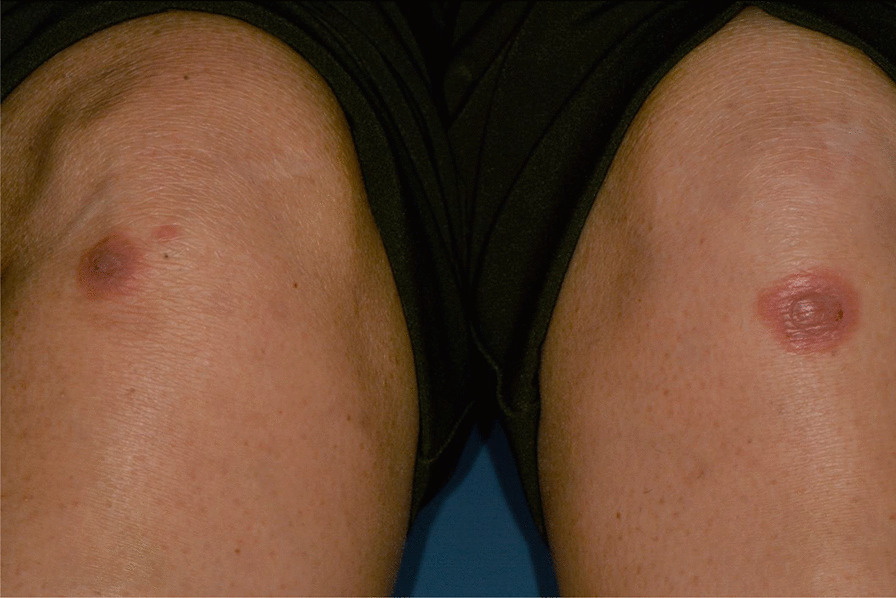
Typical erythema multiforme target lesions occurring on knees skin

**Fig. 7:**
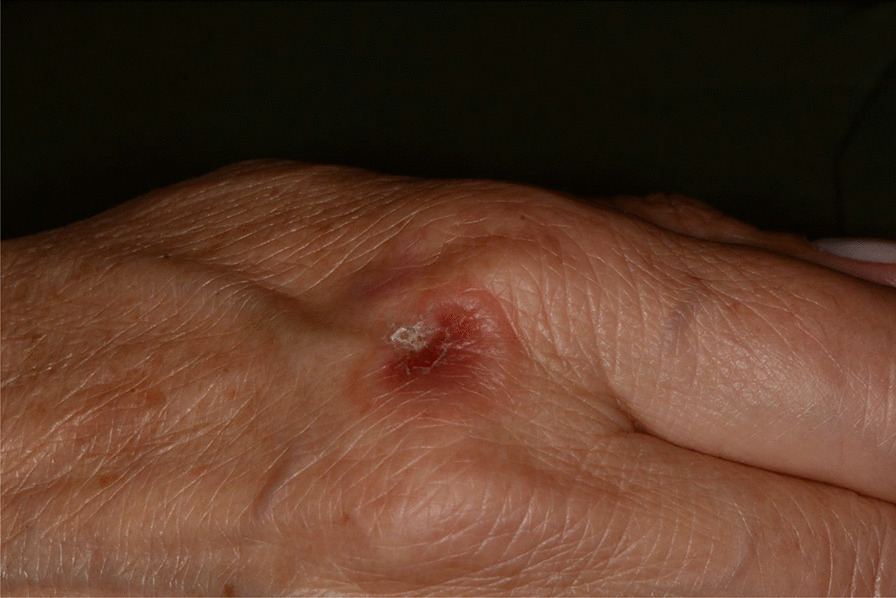
Tipical target erythema multiforme lesion on dorsal hand skin

**Fig. 8:**
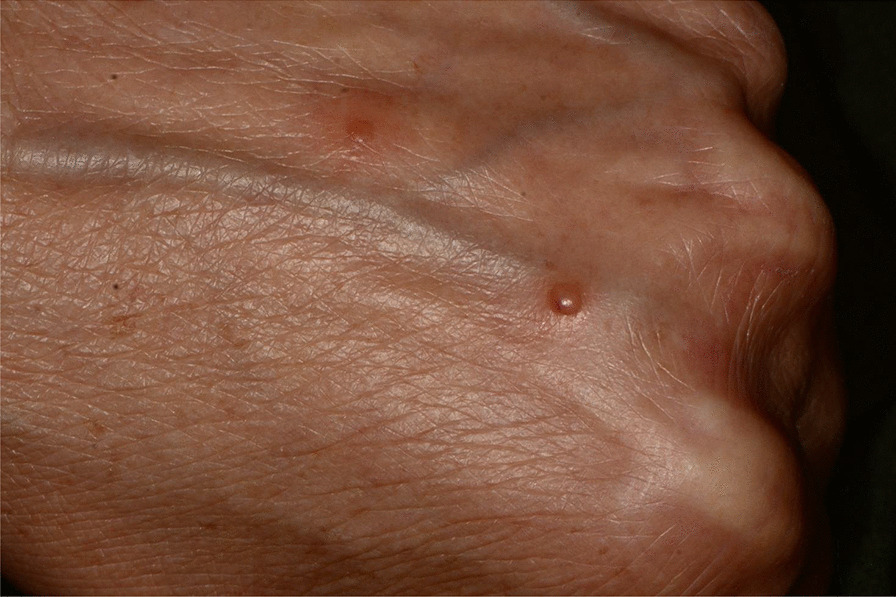
Erythematous and bullous lesions on dorsal hand skin

Later, she also developed oral mucosa’s painful erosive lesions, and concentric targetoid plaques on hands, forearms, knees, and heels (Figs. 1, 2,3, 4, 5, 6, 7 and 8). The genital mucous membranes were not involved. Before the first dose vaccination, the patient was in apparently very good health, with no recent story of herpes labialis and MMP lesions, and she was not taking any medication. Subsequently, the patient underwent a second dose of vaccine, undergoing corticosteroid administration on the advice of her general practitioner, to contain the flare’s spread, despite a mild risk of a reduced response to the vaccine [6].

The patient reported the appearance of new lesions on the hands immediately after the second dose.

She has been treated with prednisone 25 mg p.o. a day for 10 days tapering the dosage and topical 0.05% clobetasol propionate ointment. Thereafter discontinuation of corticosteroid-based therapy, a dosage of BP180 and BP230 with the enzyme-linked immunosorbent assays (ELISA) method was asked, to rule out MMP recurrence due to the medical history of the patient. The ELISA test resulted negative. The diagnosis of EM minor was posed. After 10 days of therapy, the lesions disappeared without reoccurrences.

### Case 2

**Fig. 9:**
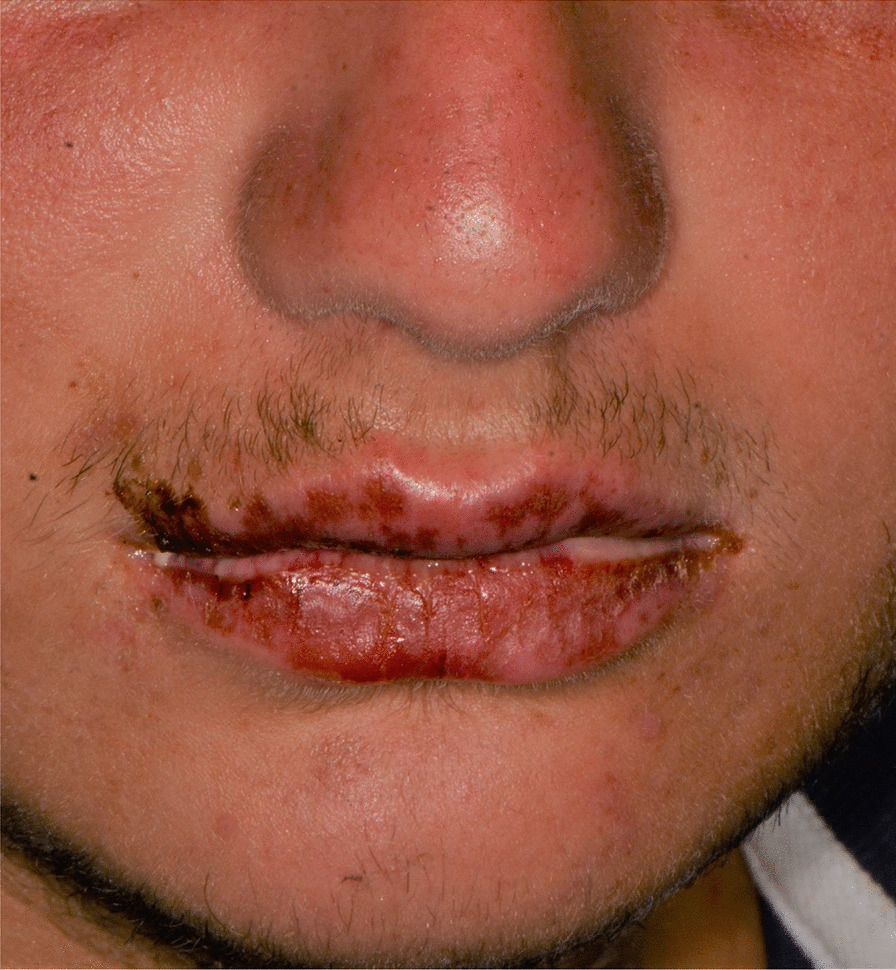
Erythematous and squamous crusted lesions on lips mucosa and vermilion

**Fig. 10:**
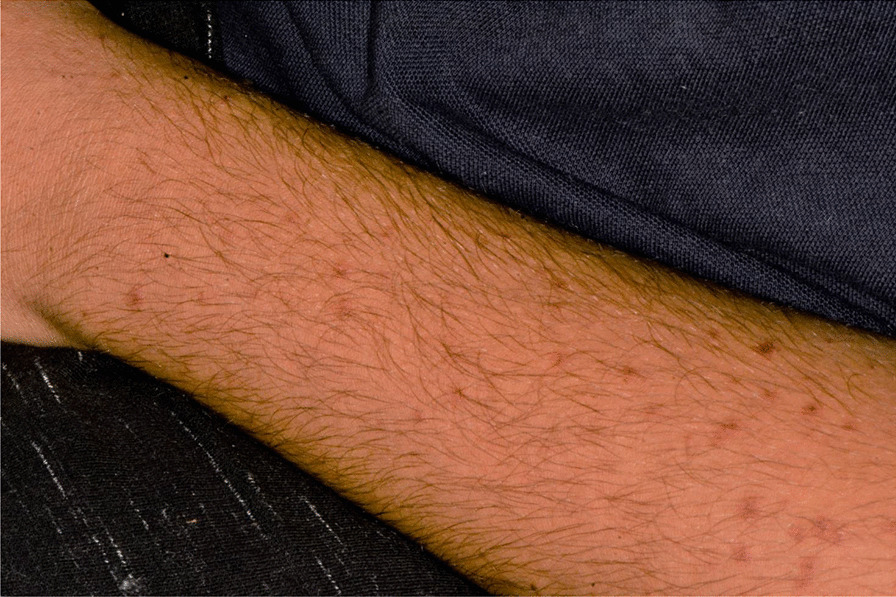
Multiple erythematous skin lesions

A 15 years-old man suffering from West Syndrome, an epilepsy form associated with cognitive impairment, developed oral erythema, erosions, and pseudo-membranes spread throughout the oral mucosa, associated with severe pain and dysphagia, 7 days after the first dose of BNT162b2 administration (Fig. 9). Multiple cutaneous lesions consisting of erythematous plaques affecting the whole body, in the particular on the trunk, legs, and neck, were also present (Fig. 10). The clinical picture was consistent with an EM minor diagnosis.

The patient did not have a medical history of cutaneous autoimmune nor herpetic diseases and he did not report any change in his usual epilepsy medical treatment, consisting of valproate.

The patient’s general practitioner prescribed him 4 mg of betamethasone and antihistamine medication, but after 10 days from the first dose vaccination he showed no improvement of his clinical condition.

**Fig. 11:**
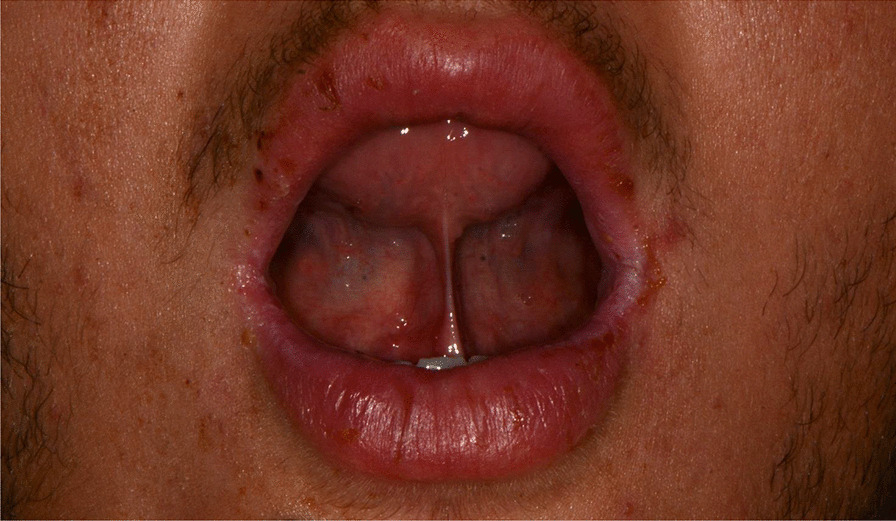
Improvement of labial and mucosal lesions after prednisone treatment

When the patient presented to our attention, he was prescribed with prednisone 25 mg p.o. a day tapering the dosage, in association to topical 0.05% clobetasol propionate ointment. After a week of treatment, the patient showed regression of mucosal and cutaneous lesions (Fig. 11).

### Case 3

A 49 years-old female came to our attention complaining of an intraoral burning sensation with a later appearance of erythematous and bullous-like lesions on the tongue and the mouth floor, arising 24 h after the second dose of BNT162b2 vaccine administration.

The patient did not refer a previous history of herpetic manifestation nor other diseases, neither she was assuming medications before the vaccination.

**Fig. 12:**
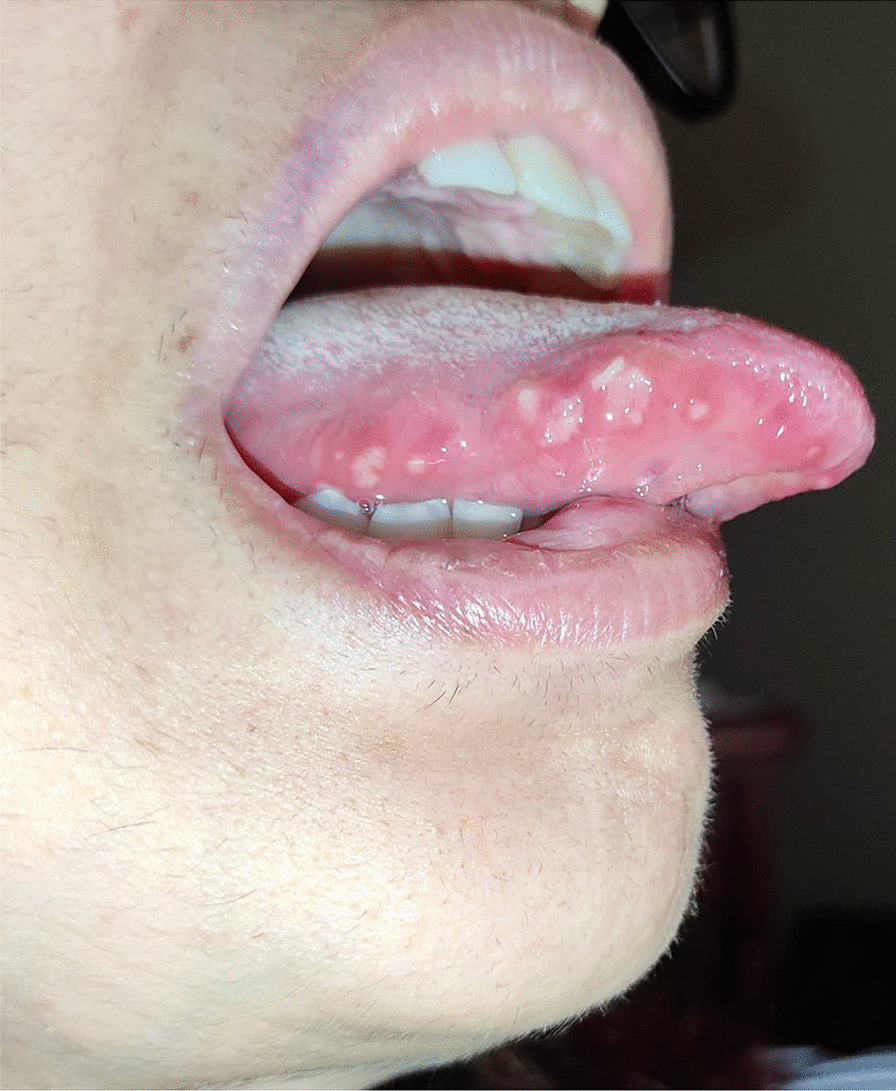
Multiple bullous and erythematous lesions on the ventral tongue

**Fig. 13:**
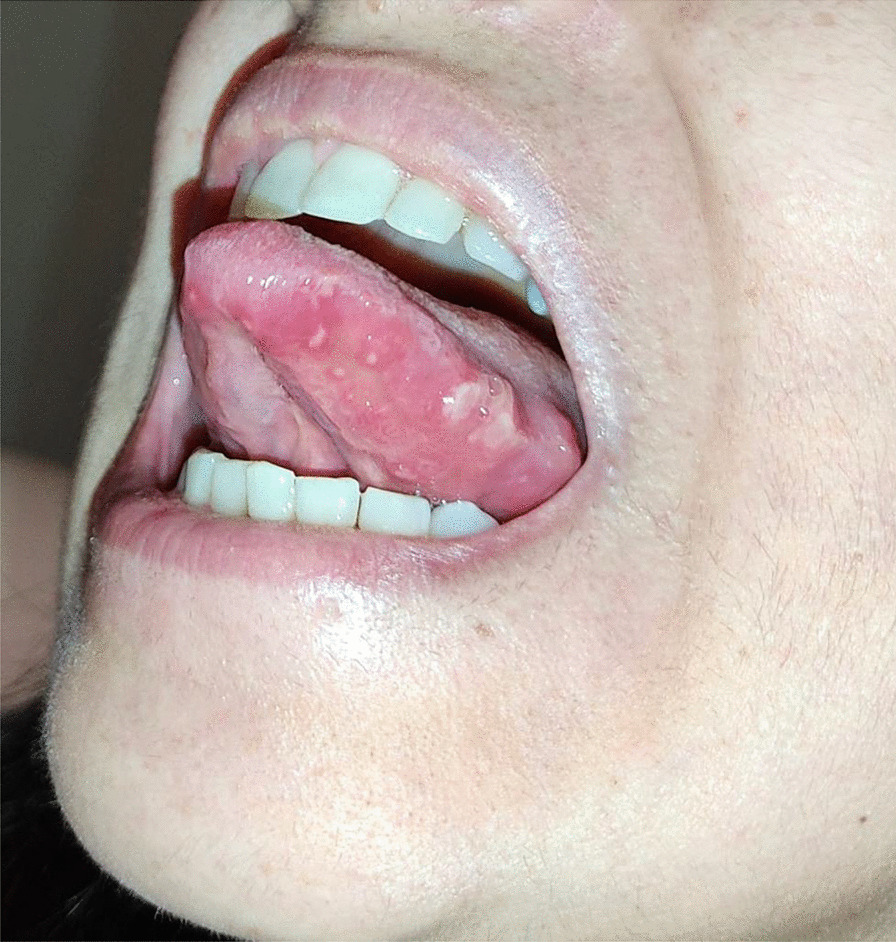
Multiple bullous and erythematous lesions on the ventral tongue

After a week the lesions presented a spontaneous improvement, but then erythematous lesions spread on the dorsal tongue, mouth floor, gingival mucosae, buccal mucosa, and soft palate arose, causing severe dysphagia (Figs. 12, 13).

No cutaneous lesions were noted, suggesting an EM minor diagnosis. A prednisone 25 mg p.o a day with tapering the dosage was prescribed for 10 days, in addition to topical 0.05% clobetasol propionate ointment. After the treatment, the lesions healed with no reoccurrence.

### Case 4

**Fig. 14:**
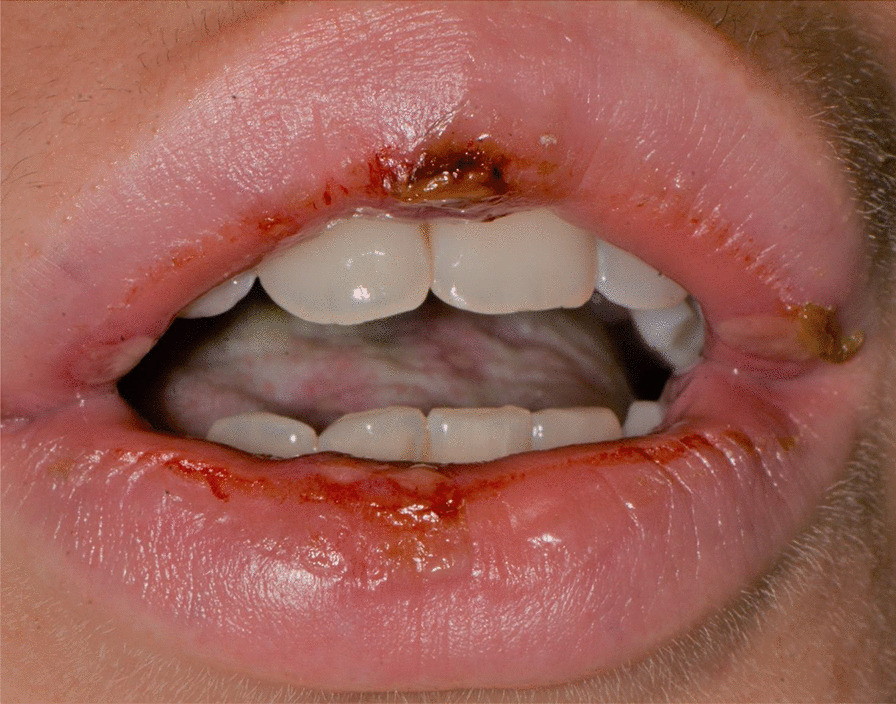
Squamous-crostous and bullous lesions on the lip’s mucosa

**Fig. 15:**
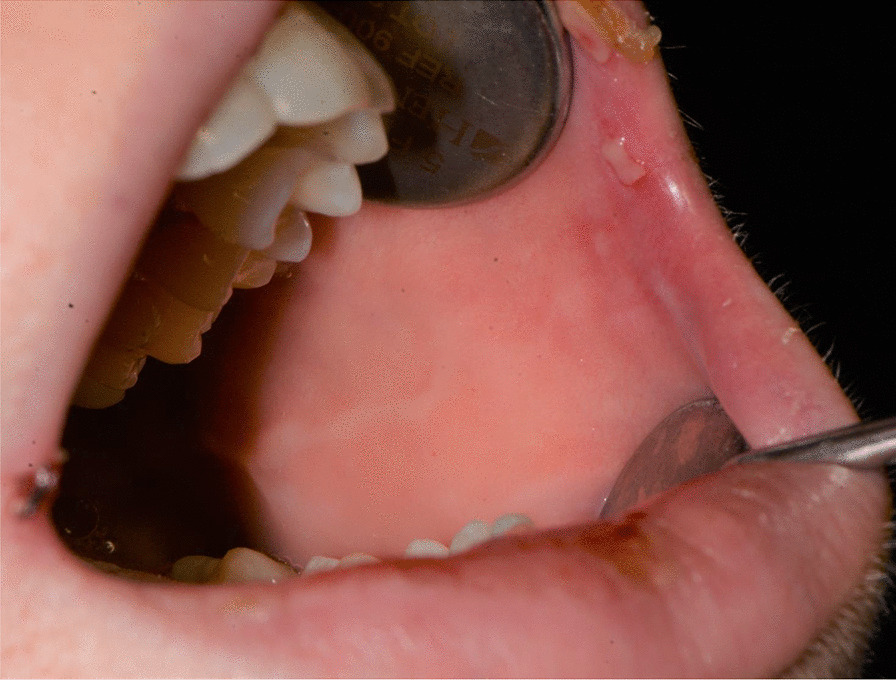
Bullous and erythematous lesions on the cheek and inner lip’s mucosa

**Fig. 16:**
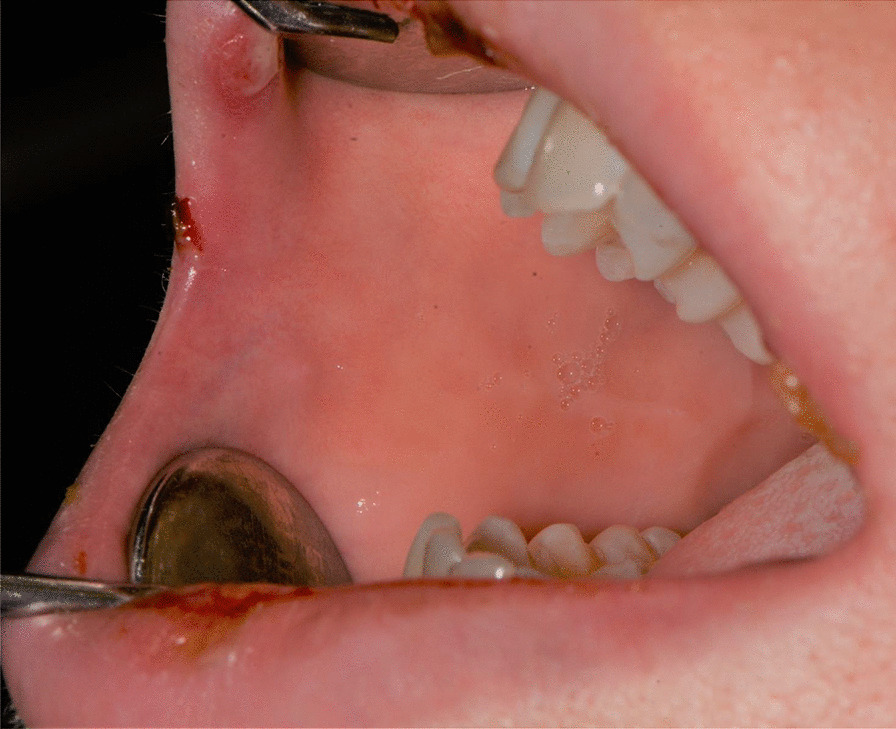
Bullous and erythematous lesions on the cheek and inner lip’s mucosa

**Fig. 17:**
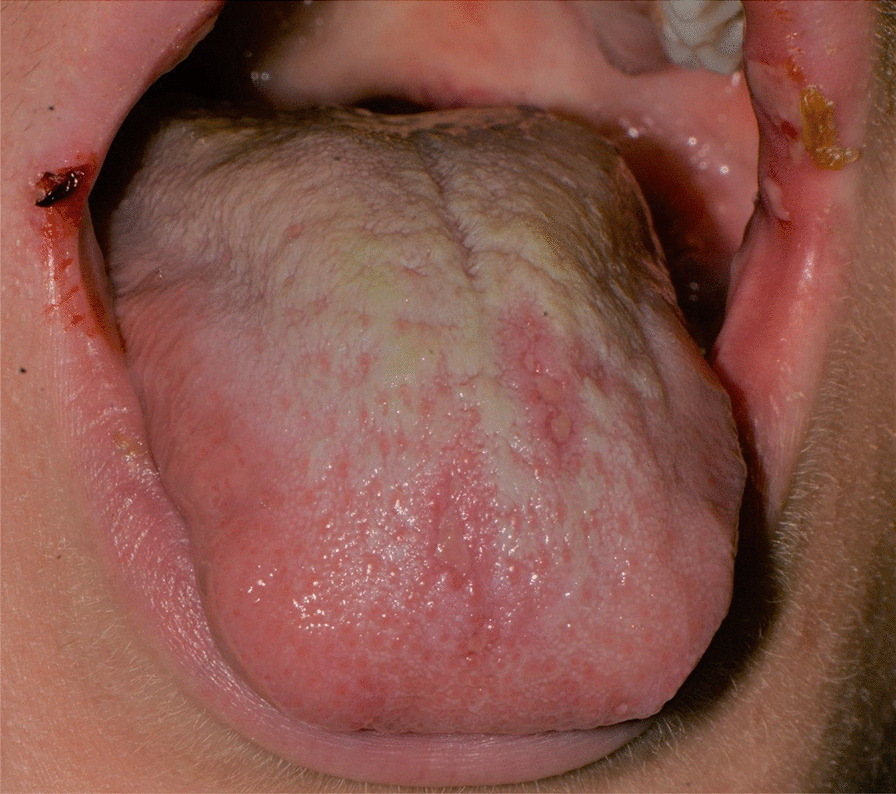
Erythematous lesions on the dorsal tongue mucosa

**Fig. 18:**
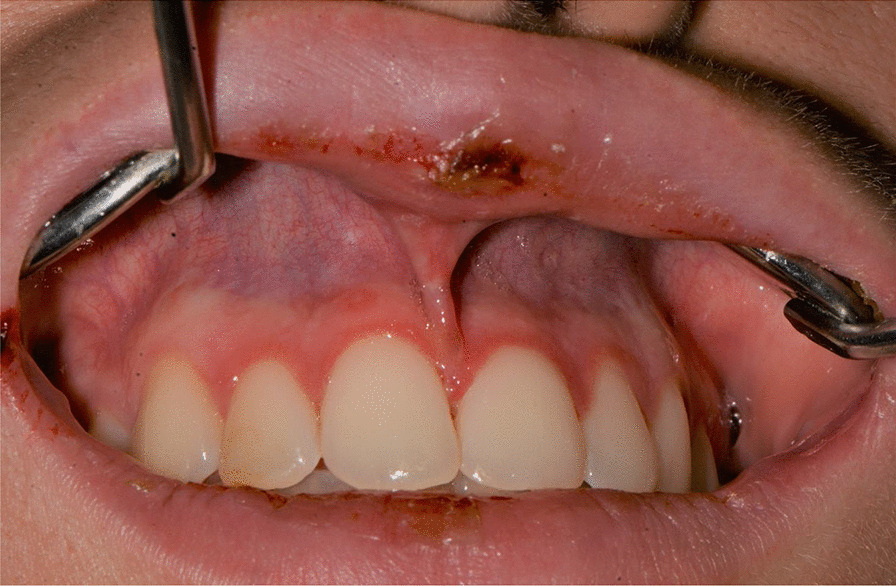
Erythematous-bullous lesions on the gingival mucosa

**Fig. 19:**
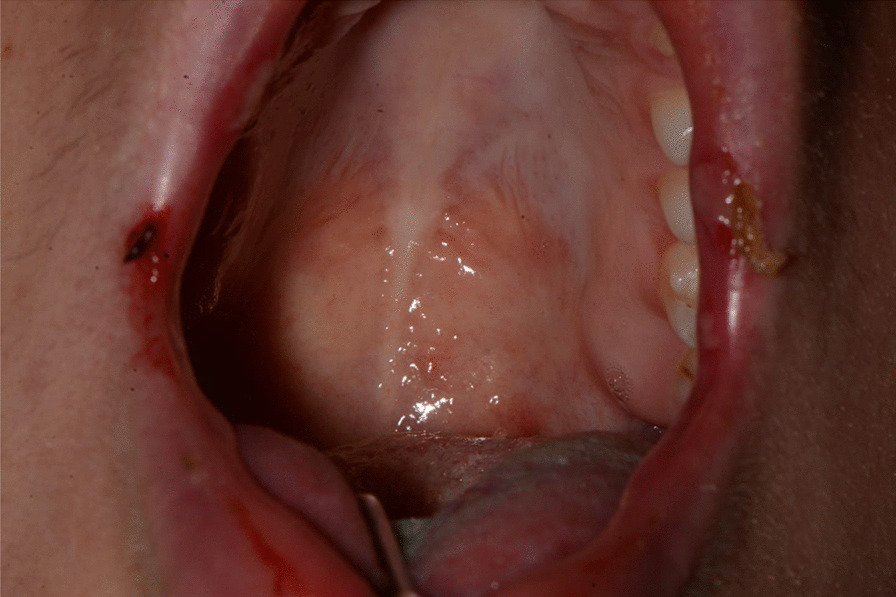
Erythematous lesions on the soft palate

A 20-years-old woman affected by celiac disease, lactose intolerance, and spastic colon, was referred to our attention 18 days after the first dose of BNT162b2 vaccine administration, complaining of pain and bleeding from the mouth, dysphagia, and dysphonia. The oral examination revealed the presence of erosions on the gingival and lips mucosa, squamous crusted lesions on labial skin, and vermillion associated with persistent fever (39 °C) (Figs. 14, 15, 16, 17, 18 and 19).

No cutaneous lesions were detected. She has already been taking valaciclovir and nystatin for 7 days without improvements. Covid-19 infection was excluded following several negative nasopharyngeal swabs. Prednisone 25 mg p.o. tapering dosage was prescribed for 3 weeks.

## Discussion and conclusions

The reported cases showed the arose of EM flare with mucous and cutaneous involvement within 10 days since receiving Pfizer/BioNTech COVID-19 vaccine administration.

None of the patients had history of EM, Stevens-Johnson syndrome, nor recurrent herpes simplex virus flare. All patients denied the employ of medication linkable to the EM manifestations.

From December 2020 to February 2021, 414 cases of dermatologic adverse reactions of the mRNA Covid-19 vaccines were reported. Among these, only 17% occurred after the Pfizer/BioNTech Covid-19 vaccine. Most of the reactions were confined to the site of the injection and occurred as urticaria (0.2%), injection site wheal (0.2%), morbilliform rash (0.1%). The symptoms usually arose within 7 days from the injection: local manifestations appeared within 1–3 days from the inoculation while systemic reactions were noted 7–8 days after vaccination [4].

The percentage of patients presenting with a delayed generalized cutaneous reaction (56%) showed also local site vaccination rash. Among them, 8.6% had varicella-zoster, 3.4% herpes simplex, 3.5% pityriasis-like manifestations. The flare of pre-existing lichen planus following BioNTech Covid-19 vaccination was reported too [7]. More than 90% of cutaneous adverse reactions appeared in women in an age between 47–56 years old, and 75% of skin reactions were reported in patients with a previous dermatological history [4].

To date, only one case of EM after BNT162b2 vaccination has been reported [8] while 3 cases are known after other mRNA vaccine’s first dose administration [4].

Lavery et al. reported a case of recurrence of EM after BNT162b2 vaccination in a female patient who was already affected by the disease in 2018, and who recurrently suffered from herpes labialis. The authors reported the occurrence of erythematous plaques on hands and feet bilaterally, but the absence of mucous membrane involvement [9].

EM also represents a possible mucocutaneous implication of Covid-19 itself, especially in children. The histological findings of these lesions highlighted the presence of SARS-COV-2 proteins in the endothelial capillary cells and epithelial cells too, together with a massive CD4 + T helper lymphocytic vasculitis and dermal inflammation. Few cases of EM linked to Covid-19 were reported in adults [9–11], sometimes presenting as the first manifestation before respiratory symptoms and signs [12].

Pathophysiological mechanisms behind the observed lesions could be related to a lymphocyte cell-mediated hypersensitivity reaction with pro‐inflammatory cytokines production targeting SARS‐ COV‐ 2 antigens located in the skin. The Covid-19 infection is usually followed by a massive inflammatory response and a huge pro-inflammatory cytokines release, defined as “cytokine storm”, due to a hyper-immune response by the host which directly causes multi-organ injury and eventually failure [13].

The vaccination could lead to the outbreak of EM due to the expression of an antigen on keratinocyte, causing T-cell activation; a mechanism already described for other vaccinations (tetanus and diphtheria, inactivated polio and Hemophilus b, smallpox, hepatitis B vaccine) [14, 15].

The EM could be also incidental to herpes simplex infection reactivation, which is a possible reaction to the BNT162b2 vaccine.

The pathogenesis of herpes-associated EM is consistent with a delayed-type hypersensitivity reaction. Reactivation of HSV-1 can be related to a failure of CD8 + T cell to maintain latency. On the other hand, vaccination stimulates the immune system and polarizes T cells [16].

Sahin et al. reported that the BNT162b2 vaccine induced a coordinated humoral and cellular adaptive immunity [16]. Seven days after the first dose, a strong cellular response with spike-specific CD8 + T cell and T helper type 1 (Th1) CD4 + T cells is expanding with a production of interferon-γ (IFNγ), a cytokine responsible for several antiviral responses [17].

Protein spike (S1)-binding antibody present after the first dose, responses increased following the second dose; significant neutralizing antibodies (Nab) was only present after the second dose [18, 19].

Spike-specific CD8 + T cell responses correlated positively with S1-binding IgG, indicating a convergent development of the humoral and cellular adaptive immunity.

These CD8 + T cells’ cluster has a phenotype with low expression of CCR7 and CD45RA and high expression of CD28 and CD27. CD8 + T cells also express markers associated with akin activation, such as CD38, HLA244 DR, and PD-1 [17].

HSV-1 establishes a latent infection due to the persistence of CD8 + T cell around infected neuronal and ganglia infected cells lifetime. CD69, CD44, CD25, and CD49d markers are expressed on activated CD8 + T cells surface. They downregulate homing receptors CD62L and CCR7 and CD27 and CD28 markers of naïve CD8 + T cells.

An excess of signals in either direction could negatively affect the T cell population, leading to exhausted effectors or dysfunctional memory [17].

During the HSV-1 latency, CD8 + T cells inhibit the gene expression of ICPO and ICP4 by the production of IFN-γ, granzymes A and B. A dysregulation of this regulation due to stress or hormonal changes may increase the expression of the lytic genes, causing assemblage of viral components and the viral release with a clinical reactivation of the infection. These CD8 + T cells never leave the trigeminal ganglia: they survive in the tissue without being replenished from circulating pools of CD8 + T cells and maintain an homeostatic turn-over [16].

Several possible mechanisms whereby viral genome copy number might influence reactivation from the latent state can be envisioned. Many viral genome copies could effectively overwhelm factors silencing viral gene transcription, leading to an increased probability of reactivation.

However, these new cases do not prove causality, and extensive epidemiological or experimental studies are needed to highlight the possible link between vaccination and EM, through autoimmune cross-reactivity due to reactivation of the herpes virus (HSV-1).

## Data Availability

Data sharing is not applicable to this article as no datasets were generated or analyzed during the current study.
